# Choroiditis following severe acute respiratory syndrome coronavirus 2 infection in unvaccinated identical twins

**DOI:** 10.5935/0004-2749.2023-0117

**Published:** 2024-07-09

**Authors:** Luiz Guilherme Marchesi Mello, Lara Guedes Lubiana, Carlos Eduardo Hirata, Mário Luiz Ribeiro Monteiro, Joyce Hisae Yamamoto

**Affiliations:** 1 Division of Ophthalmology and Laboratory of Investigation in Ophthalmology (LIM-33), Faculdade de Medicina, Universidade de São Paulo, São Paulo, SP, Brazil; 2 Department of Specialized Medicine, Centro de Ciências da Saúde, Universidade Federal do Espírito Santo, Vitória, ES, Brazil; 3 Ophthalmology Unit, Hospital Universitário Cassiano Antonio Moraes, Empresa Brasileira de Serviços Hospitalares, Vitória, ES, Brazil

**Keywords:** Choroiditis, Coronavirus, COVID-19, SARS-CoV-2, Uveitis

## Abstract

Unvaccinated identical twins developed bilateral anterior uveitis soon after the onset of coronavirus disease 2019 symptoms. During follow-up, both patients developed choroiditis, and one twine developed posterior scleritis and serous retinal detachment. Prompt treatment with oral prednisone ameliorated the lesions, and no recurrence was observed at the 18-month follow-up. Choroiditis may rarely be associated with severe acute respiratory syndrome coronavirus 2 infection, and it responds well to corticosteroid therapy. Although the exact mechanism is unknown, we hypothesize that the virus may act as an immunological trigger for choroiditis.

## INTRODUCTION

The severe acute respiratory syndrome coronavirus 2 (SARS-CoV-2) was associated with the outbreak of the coronavirus disease 2019 (COVID-19) in late 2019, which led to a pandemic in early 2020 and continues to be a health concern. The spectrum of the disease, primarily respiratory, ranges from an asymptomatic or oligosymptomatic infection to severe acute respiratory syndrome^(1)^. It can also affect other organs and tissues such as the central nervous system and eyes^(2)^. Except for conjunctivitis, ocular manifestations related to SARS-CoV-2 are uncommon^(3)^. Reports of choroidal lesions in patients with confirmed COVID-19 are rare^(4,5,6,7,8)^. Herein, we have reported the first instance of identical twins who developed choroiditis following SARS-CoV-2 infection confirmed by real-time reverse transcription polymerase chain reaction (RT-PCR). Additionally, one of them developed posterior scleritis.

## CASE REPORTS

A 17-year-old Brazilian boy (twin A) presented with complaints of redness, photophobia, and mild pain in the right eye (OD) since 1 month. He reported flu-like symptoms one week before the onset of ocular symptoms and was diagnosed with COVID-19, which was confirmed via the RT-PCR results of nasopharyngeal swabs. His medical history was otherwise unremarkable and he denied being vaccinated against COVID-19 or any other disease.

On examination, his visual acuity (VA) was 20/20 in both eyes (OU), and the pupillary, ocular motility, and intraocular pressure evaluations were normal. There were fine keratic precipitates in OU, 2+ and 1+ anterior chamber cells in the OD and left eye (OS), respectively, as well as a mild (trace) anterior vitreous reaction in OU. A fundoscopic examination of OU was unremarkable. The patient was diagnosed with bilateral non-granulomatous anterior uveitis and was started on 1% prednisolone eyedrops, to be instilled four times a day in OU. The dose was gradually tapered weekly.

The patient reported that his identical twin (twin B) had also been diagnosed with COVID-19, which was confirmed by RT-PCR, 1 week after him. Twin B also complained of the same ocular symptoms and under­went a complete ophthalmologic examination. He revealed that he had developed a right-sided Bell’s palsy 2 years ago, without any sequelae, and denied being vaccinated against COVID-19. His VA was 20/20 in OU, and the pupillary, ocular motility, and intraocular pressure evaluations were normal. There were fine keratic precipitates in OU, 3+ and 2+ anterior chamber cells in the OD and OS, respectively, and a mild anterior vitreous reaction in OU. The fundoscopic examination was unremarkable. Twin B was also diagnosed with bilateral non-granulomatous anterior uveitis. He was started on 1% prednisolone eye drops every hour in OU, and the dose was tapered weekly.

Laboratory blood investigations of both patients revealed a normal complete blood count, C-reactive protein level, and erythrocyte sedimentation rate. Furthermore, their urine analyses and chest computed tomography reports were also normal. Tests for IgG and IgM against cytomegalovirus, herpes simplex virus (IgG was positive in twin B), varicella-zoster virus, Epstein-­Barr virus, and toxoplasmosis were negative. Additionally, tests for FTA-ABS, VDRL, anti-HIV, anti-HCV, HBsAg, anti-HTLV-1/2, antinuclear antibodies, rheumatoid factor, and perinuclear and classical antineutrophil cytoplasmic antibodies were negative, as was the purified protein derivative test (0 mm).

**Figure 1:**
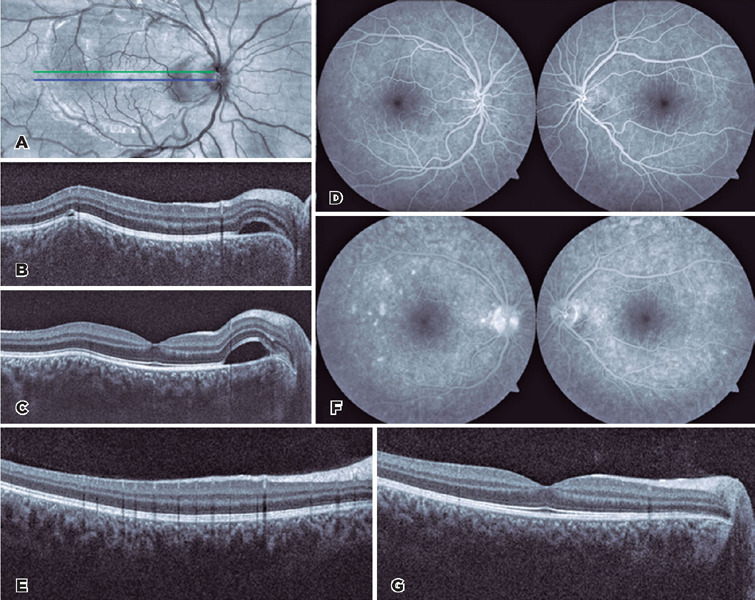
Multimodal fundus imaging in twin B. Pretreatment (A) infrared fundus imaging of the right eye and optical coherence tomography (OCT) at panel A’s (B) green and (C) blue arrow regions revealed a serous retinal detachment in the peripapillary region and another smaller one in the temporal region above a localized elevated choroidal lesion. (D) Early and (E) late phases of fluorescein angiography confirmed this. Post-treatment OCT images at the same regions as that of (F) panel B and (G) panel C revealed resolution of the abnormalities.

The anterior uveitis gradually improved over the next three weeks in both twins. However, at the 4^th^-week follow-up, twin B complained of severe ocular pain in the OD, which worsened with eye movements, and mild ipsilateral visual blurring. His VA remained 20/20, and the pupillary reactions, ocular motility, and intraocular pressure in OU were normal. The OD examination revealed 0.5+ anterior chamber cells and mild anterior vitreous reaction. The fundoscopic evaluation revealed a yellowish-white rounded choroidal lesion temporal to the macula in the OD and a serous retinal detachment around the optic disc. A smaller choroidal lesion was noted temporal to the macula in the OS, as evidenced by optical coherence tomography (OCT) (Figure 1A-C). Ultrasonography of the OD revealed a T-sign and fluorescein angiography demonstrated small points of hyperfluorescence in the region of the lesion in OU (Figure 1D-E).

**Figure 2:**
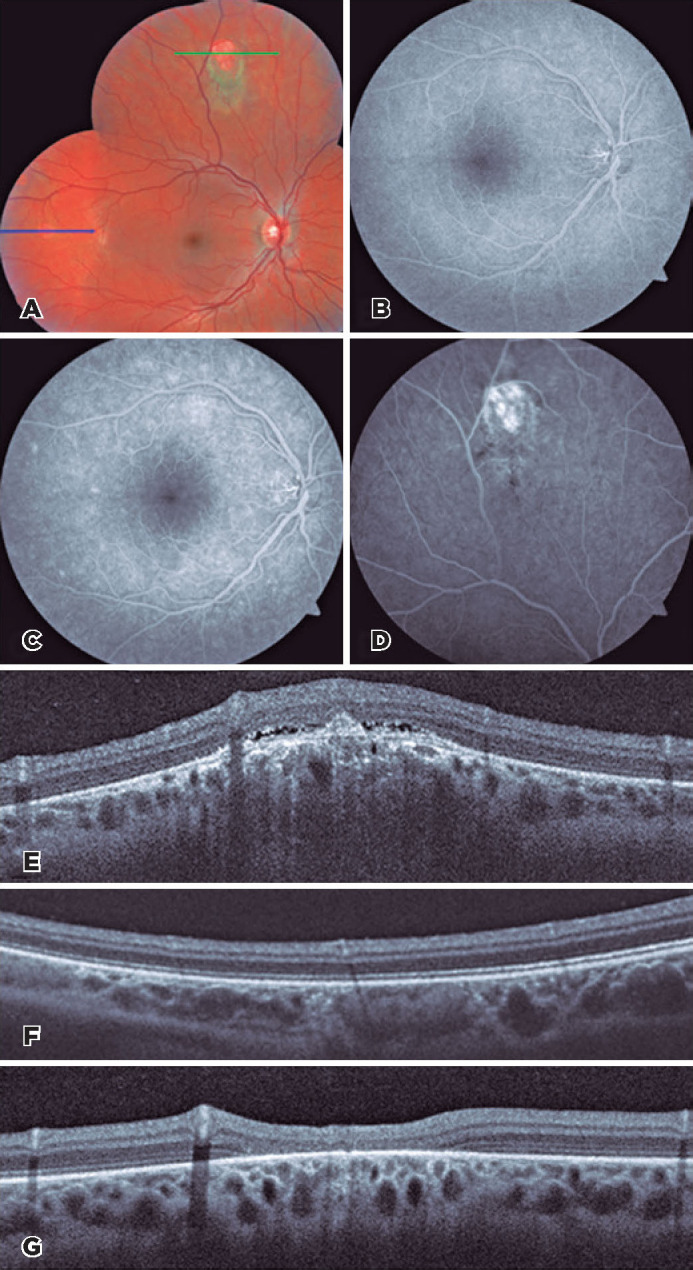
Multimodal fundus imaging of the right eye in twin A. Pretreat­ment montage of (A) color fundus images and (B) early and (C) late phases of fluorescein angiography showing the macular area. (D) The superior lesion on fluorescein angiography. Optical coherence tomography (OCT) images at panel A’s (E) green and (F) blue arrow regions revealed a small serous retinal detachment, focal disruption of the outer retinal layers, and retinal pigment epithelium mottling above a localized elevated choroidal lesion. (G) Post-treatment OCT at the same region as that of the green arrow in panel A revealed outer retinal atrophy and improvement in the choroidal thickening.

Although twin A was asymptomatic, a complete oph­thalmologic examination was performed. There was an improvement in the number of anterior chamber cells (trace) in OU. However, the fundoscopic examination revealed three choroidal lesions in the OD, which were confirmed by OCT and fluorescein angiography (Figure 2A-F). Oral prednisone (0.8mg/kg/day for twin B and 0.5mg/Kg/day for twin A) was administered and tapered weekly over three months. Three weeks later, the lesions had completely resolved in both twins, except for the larger superior lesion in the OD of twin A, which had formed a scar (Figure 1F-G and Figure 2G).

The twins did not develop any recurrence of uveitis after an 18-month follow-up, even after the administration of COVID-19 vaccine after a 6-month follow-up without corticosteroid therapy. Two doses of inactivated SARS-CoV-2 vaccine (CoronaVac®, Sinovac Biotech, Beijing, China) were administered at an interval of 1 month, and a booster dose of the ChAdOx1-S/nCoV-19 vaccine (AZD1222, AstraZeneca, Cambridge, United Kingdom) was administered four months after the second dose.

## DISCUSSION

More than two years have passed since the onset of the COVID-19 pandemic. However, there is still uncertainty regarding the ophthalmologic manifestations associated with SARS-CoV-2 infection and the possible role of the virus as an immunological trigger^(2,3,4)^. Based on the inflammatory choroidal lesions and extensive etiological investigations performed in our patients, the diagnostic hypothesis was multifocal choroiditis associated with systemic SARS-CoV-2 infection. Concurrent choroiditis and posterior scleritis were also observed in one twin (twin B).

Although the possibility of direct choroidal invasion cannot be completely ruled out, we believe that SARS-­CoV-2 triggered the inflammatory response and led to the development of choroidal lesions. The bilateral involvement in the identical twins, posterior uveitis 3 weeks after anterior uveitis, and marked improvement of the choroidal lesions after corticosteroid therapy suggest a possible immune-mediated mechanism.

Some studies suggest a possible genetic relationship between COVID-19 susceptibility and disease severity^(9,10,11)^. Angiotensin-converting enzyme polymorphisms, high-risk human leukocyte antigen haplotypes, the ABO locus, and several genes of cellular proteases, androgen receptors, and interferons are some of the genetic risk factors associated with COVID-19^(10)^. In our patients, a genetic analysis was not performed. Thus, we cannot exclude a possible association between a genetic risk factor, a COVID-19 infection, and the uncommon ophthalmological manifestations in the identical twins.

Vaccines against COVID-19 were authorized worldwide in healthcare systems in December 2020, and Brazil started vaccinating priority groups in January 2021. Pediatric use of the vaccine was authorized later in the same year^(12)^. At the time of disease onset, our patients were unvaccinated because they did not meet the Brazilian public health system criteria, making vaccination an unlikely confounding factor. At a 6-month follow-up after discontinuing corticosteroid therapy, the patients were vaccinated without recurrence of uveitis, despite the risk of reactivation of inflammation. However, patients should be closely followed up, especially in cases of chorioretinal scarring, as seen in twin A.

In this study, we have reported an association between anterior uveitis and SARS-CoV-2 infection in addition to the development of choroiditis, scleritis, and focal serous retinal detachment in two identical unvaccinated twins. Systemic steroid treatment induced a complete resolution, and no relapses occurred even after being vaccinated against COVID-19. Our findings emphasize the need for close follow-up and complete ophthalmologic examination in patients with recent previous SARS-­CoV-2 infection and any ophthalmological symptoms.
